# Molecular Insights of Cholestasis in MDR2 Knockout Murine Liver Organoids

**DOI:** 10.1021/acs.jproteome.3c00900

**Published:** 2024-03-15

**Authors:** Irene Blázquez-García, Laura Guerrero, Cristina Cacho-Navas, Nabil Djouder, Jaime Millan, Alberto Paradela, Lorena Carmona-Rodríguez, Fernando J. Corrales

**Affiliations:** † Functional Proteomics Laboratory, Centro Nacional de Biotecnología (CSIC), Madrid 28049, Spain; ‡ Centro de Biología Molecular Severo Ochoa (CBMSO), Madrid 28049, Spain; § Centro Nacional de Investigaciones Oncológicas (CNIO), Madrid 28029, Spain

**Keywords:** organoid, MDR2, PFIC3, cholestasis and hepatocellular carcinoma

## Abstract

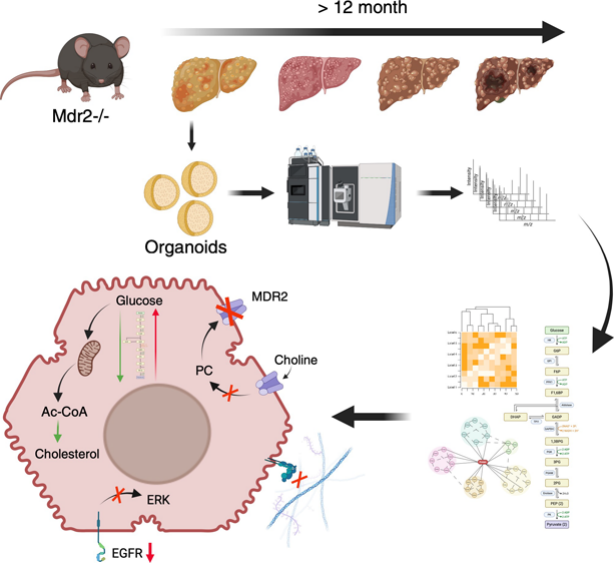

MDR3 (multidrug resistance 3) deficiency in humans (MDR2 in mice) causes progressive familial intrahepatic cholestasis type 3 (PFIC3). PFIC3 is a lethal disease characterized by an early onset of intrahepatic cholestasis progressing to liver cirrhosis, a preneoplastic condition, putting individuals at risk of hepatocellular carcinoma (HCC). Hepatocyte-like organoids from MDR2-deficient mice (MDR2KO) were used in this work to study the molecular alterations caused by the deficiency of this transporter. Proteomic analysis by mass spectrometry allowed characterization of 279 proteins that were differentially expressed in MDR2KO compared with wild-type organoids. Functional enrichment analysis indicated alterations in three main cellular functions: (1) interaction with the extracellular matrix, (2) remodeling intermediary metabolism, and (3) cell proliferation and differentiation. The affected cellular processes were validated by orthogonal molecular biology techniques. Our results point to molecular mechanisms associated with PFIC3 that may drive the progression to liver cirrhosis and HCC and suggest proteins and cellular processes that could be targeted for the development of early detection strategies for these severe liver diseases.

## Introduction

Cholestasis is a liver syndrome that results from an impaired bile formation or flow that leads to the accumulation of bile components and concomitant cellular and tissular damage. The clinical presentation of cholestasis is diverse, ranging from mild to severe symptoms involving inflammation, fibrosis, cirrhosis, and ultimately hepatocellular carcinoma (HCC) or cholangiocarcinoma. Several etiological determinants are associated with cholestasis, including genetic defects that are especially relevant in children.^1^ Progressive familial intrahepatic cholestasis (PFIC) is an autosomal recessive disease that belongs to the group of rare liver diseases, with an estimated prevalence of 1 case per 50,000–100,000 births. PFIC commonly presents during childhood and might lead in the short term to severe liver injury and cancer in the first decade of life. PFIC groups have been defined according to the gene carrying the mutation: PFIC1 or Byler’s disease, whose defective gene is *ATP8B1* that encodes the amino-phospholipid flippase FIC1; PFIC2 which results from the expression of BSEP variants encoded by mutated versions of *ABCB11* gene and PFIC3, that results from mutations in the *ABCB4* gene encoding the MDR3 protein (multidrug resistance protein 3; MDR2 in mice).^2^

PFIC3 is a lethal disease characterized by an early onset of cholestasis, liver cirrhosis, and end-stage liver disease, at risk of HCC, with life-threatening consequences without a liver transplant.^3^ About 300 ABCB4 variants have been described, which encode different MDR3 protein forms with different degrees of functional impairment. MDR3 protein is a liver-specific phosphatidylcholine (PC) transporter located in the canalicular membrane of hepatocytes.^4^ It is a p-glycoprotein that integrates six extracellular loops separated by 12 transmembrane domains and two intracytoplasmic ATP-binding domains (NBDs). MDR3 is an energy (ATP)-dependent flipase that transports PC from the cellular interior to the bile canaliculus. Then, cholesterol–PC vesicles and mixed bile acid micelles are formed to neutralize the detergent effect of hydrophobic bile acids during transport through the biliary network. Impaired MDR3 activity prevents the formation of micelles, leaving free bile salts that induce an epithelial injury and initiate the pathogenic process. Concomitantly, the absence of phospholipids in the bile also prevents the maintenance of biliary cholesterol in solution, leading to the formation of cholesterol crystals and inducing biliary lithiasis. These early alterations lead to the obstruction of the bile ducts and to the presentation of the most common symptoms, such as pruritus, fatigue, abdominal pain, jaundice, elevation of aminotransferases, and in some cases GGT and alkaline phosphatase.^5^

Despite the heavy societal burden represented by PFIC3, therapeutic options for patients with PFIC are currently very limited. In cases with residual MDR3 activity, ursodeoxycholic acid (UDCA) has shown a positive effect. UDCA is a hydrophilic bile acid, nontoxic to hepatocytes, that is able to replace cell-damaging hydrophobic bile acids as well as to stimulate the expression of MDR3 (in PFIC3) and BSEP (in PFIC2) by increasing phospholipid secretion.^2^ However, it has been reported that for many children with a complete absence of MDR3 function, the efficiency of this treatment is limited and does not prevent the progression of a deleterious liver disease. For such cases, liver transplantation remains the only therapeutic option for 75–100% of patients in their 3–5 years of age.^6^ Nevertheless, the scarcity of young liver donors and the permanent immunosuppression therapy required by patients after transplantation represent significant challenges for the clinical management of PFIC3 patients. Systematic investigation of the mechanisms underlying the onset of PFIC3 would shed light on the molecular drivers, providing valuable clues to define innovative therapeutic/palliative strategies.^4^

To study in detail the molecular basis of PFIC3, an MDR2-deficient mouse model (MDR2KO) was developed. The MDR2 deficiency reproduces part of the pathogenic factors underlying the progression of PFIC3 in mice, including inflammation and fibrosis.^6^ Moreover, the MDR2KO model has been used to test innovative therapies to combat PFIC3. In this regard, it has been demonstrated that the combined administration of tolerogenic nanoparticles containing rapamycin (ImmTOR) and an adeno-associated virus expressing MDR3 (AAV8-MDR3) had a remarkable therapeutic effect, preventing the progression of the disease.^7^ Preclinical nonanimal models have also been developed in the past few years that allow for an efficient and detailed analysis of cell biology and become useful tools to identify new druggable targets and test novel drug candidates. In particular, organoid cultures allow to reproduce tissue-specific disease models since organoids partially reproduce the complexity of the 3D organization of tissues and may trigger responses to external stimuli more closely than 2D cell cultures, avoiding the use of animal models that fail to accurately model several aspects of human development and disease.^8^ Liver organoids have been recently produced from adult liver stem cells by providing the appropriate extracellular matrix (ECM) environment and an optimized cocktail of growth factors that resemble the stem cell niche within the tissue.^9^

To further explore the early events involved in the progression of cholestasis in PFIC3 that we have recently described,^10^ we characterized the changes in the proteome of MDR2-deficient hepatocyte-like organoids using quantitative shotgun proteomics. Our results provide molecular mechanisms of essential cellular processes involved in the development of PFIC3 cholestasis in an immune system-free organoid model.

## Methods

### Biological Samples

Liver organoids from WT and MDR2KO (MDR2–/−) mice (FVB.129P2-Abcb4tm1Bor/J) were generated and cultured as described.^11,12^ Briefly, livers were isolated from 3 month-old WT and MDR2KO mice and digested using a mix of collagenase and Dispase II. Isolated hepatic bipotent adult stem cells were embedded in Matrigel (BD Bioscience) and cultured in expansion medium containing a cocktail of liver-specific growth factors such as EGF, FGF10, Rspo1, HGF, and nicotinamide. To generate functional hepatocyte-like cells, liver organoids were cultured for 11–13 days with growth factors that blocked Notch and TGFβ signaling responsible for biliary cell fate determination.

### Transmission Electron Microscopy

Matrigel-embedded liver organoids were in situ fixed with 4% paraformaldehyde and 2% glutaraldehyde in 0.1 M phosphate buffer, pH 7.4 for 2 h at room temperature. Postfixation was carried out with a mixture of 1% osmium tetroxide and 0.08% potassium ferricyanide for 1 h at 4 °C and then with 2% uranyl acetate for 1 h at room temperature. Samples were dehydrated with ethanol and processed for standard Epon embedding (TAAB-812). Orthogonal ultrathin sections (80 nm) were collected on Formvar-coated slot grids and stained with uranyl acetate and lead citrate. Sections were examined in a Jeol JEM-1400Flash transmission electron microscope operating at 100 kV. Images were taken with a Gatan OneView (4K × 4K) CMOS camera.

### RT-qPCR

Total RNA was extracted from mouse organoids using RNeasy Mini Kit (50) (74104, QIAGEN). Culture medium was removed without disturbing the 3D culture. Using wide bore tips, the drops of Matrigel containing the liver organoids were collected in falcon tubes and washed with PBS 1× at <100 g for 5 min. Liver organoids were carefully resuspended in 2.5 mL of cell recovery solution and incubated for 40 min at 4 °C with soft agitation. After incubation, liver organoids were washed three times in PBS 1× and lysated in 20% SDS buffer with a cocktail of protease inhibitors. cDNA strands were synthesized from 500 ng of total RNA using the high-capacity cDNA reverse transcription kit (Applied Biosystems), employing random primers. mRNA levels were quantified by qPCR in a Quant-Studio 5 Real-Time PCR System (Thermo Fisher) using the SYBR EvaGreen-based reaction mix (5x PyroTaq EvaGreen qPCR Mix Plus ROX) (Cmb-Bioline) and specific primers (Supplementary Table 1); *gapdh* was used as the housekeeping gene for normalization purposes. Relative quantities (Rq) (i.e., relative to the sample with the lowest expression or the control sample) were calculated using the 2^–ΔΔCt^ method.^13^

### Western Blot

Organoids were lysed in UTT-SDS buffer (7 M Urea; 2 M Tiourea; 0,1 M TEAB, tetraethylammonium bromide; 5% SDS, sodium dodecyl sulfate) in the presence of protease (cOmplete UltraTablets, Merck) and phosphatase (PhoSTOP, Merck) inhibitors, and soluble proteins were obtained by centrifugation (12,000 *g*, 5 min, 4 °C). DNA from total lysates was removed by sonication and centrifugation at 12000g for 10 min. Protein mixtures were resolved by 12% acrylamide/bis(acrylamide) (29/1) SDS-PAGE and transferred using 15 mM Tris, 192 mM glycin, and 20% v/v methanol onto nitrocellulose membranes (0.45 μm, 1620115, BioRad) for immunoblot analysis with antibodies (Supplementary Table 2). The resulting bands were quantified using an Alliance Q9 Advanced software (Uvitec, Cambridge). Entire Western blot membranes are shown in Supplementary Figure 5.

### Sample Preparation for LC-MS/MS Analysis

Aliquots of organoid lysates containing 50 μg of protein were loaded into micro S-Trap columns (Protifi). Reduction and alkylation of Cys sulfhydryl groups were then performed by incubation with tris (2-carboxyethyl) phosphine (TCEP, 5 mM) and chloroacetamide (CCA, 10 mM), for 1 h at 37 °C under agitation. Protein digestion was performed on the addition of trypsin (90057, Thermo Fisher Scientific) at a 1:15 ratio (μg trypsin: μg protein) o/n at 37 °C.^14^ The resulting peptide concentration was determined with a fluorometric Qubit assay (Invitrogen).

Isobaric labeling of the tryptic peptides was performed with the TMT-6plex kit (TMTsixplex Isobaric Label Reagent Set, 90057, Thermo Fisher Scientific; Supplementary Table 3). 35 μg peptides from each sample were resuspended in 100 μL of labeling buffer (50% acetonitrile, ACN/25 mM TEAB) along with its corresponding label tag. The labeling reaction was carried out for 2 h at room temperature under stirring (600 rpm). The reaction was stopped by incubation at room temperature for 15 min with 0.3% hydroxylamine. Then, all labeled samples were mixed, and an aliquot containing 80 μg of peptides was taken for peptide prefractionation. The resulting aliquots were dried in Speed-vac and stored at −20 °C.

80 μg of labeled peptides were resuspended in 100 μL 1% formic acid (FA). Solid-phase fractionation was carried out in CDS Empore SDB-RPS Extraction Disks (13-110-022, Fisher Scientific) at basic pH due to the presence of ammonium formate (NH4HCO2) and at increasing concentrations of ACN. Finally, the eluted fractions were combined together (1–6, 2–7, 3–8, 4–9, 5–10) and dried in the Speed-vac. The peptide concentration for each fraction was determined with a fluorometric Qubit assay (Invitrogen).

### LC-MS/MS Analysis

One microgram of the tryptic digest was analyzed by 1D-nano LC (Ultimate 3000 nano HPLC, Thermo Fisher Scientific) coupled to an Orbitrap Exploris 240 mass spectrometer (Thermo Fisher Scientific). Peptides were separated on an Easy-spray PepMap C18 analytical column (50 cm × 75 μm) at 45 °C by applying a flow rate of 250 nL/min with a 120-min gradient from 4 to 95% mobile phase B (Mobile phase A: 0.1% FA; mobile phase B: 80% ACN in 0.1% FA). In the last 10 min of this gradient, the ratio of each phase returns to the initial conditions (96% Phase A and 4% Phase B). The sample loading buffer was 2% ACN in 0.1% FA, and the injection volume of the equipment was 5 μL.

Data acquisition was carried out with the data-dependent acquisition (DDA) method, in positive mode, monitoring all ions in a range of 375–1200 mass/charge (*m*/*z*). The 20 most intense ions from each MS1 scan were selected and fragmented by higher-energy collisional dissociation (HCD). The resolution of each spectrum after fragmentation was set at 45,000 at 200 *m*/*z*, the precursor isolation window at 0.7 *m*/*z*, with an exclusion time of 45 s, and the HCD collision energy at 30. Precursor ions that showed one or no assignment after fragmentation were excluded as well as those with a charge of 6 or higher.

Raw mass spectrometry data were processed with the Proteome Discoverer (v2.5, Thermo Fisher Scientific) using four search engines (Mascot (v2.7.0), MsAmanda (v2.4.0), MsFragger (v3.1.1), and Sequest HT), and two databases (target/decoy) constructed from SwissProt *Mus musculus* sequences (Tax ID: 10090, 25097 sequences, 09/22/2021). Two types of searches were performed depending on the configuration of the TMT modification (+229 163 Da in lysine and N-terminal peptides): (1) Dynamic search, if this modification was set as a variable modification, which is used to calculate the efficiency of TMT tagging; (2) Static search, if it was set as a fixed modification, which is used to quantify tagged peptides. The rest of the search parameters were fixed modification, carbamidomethylation (+57 021 Da); variable modifications, Gln-pyroGlu (+17 027 Da), methionine oxidation (+15,9949 Da), N-terminal acetylation (+42 011 Da); enzyme, trypsin, and two missed cleavages allowed. Protein identification was performed considering false discovery rate (FDR) < 1% at peptide spectrum match (PSM), peptide, and protein levels. The results from the static search were used for the statistical analysis. The protein quantification values were calculated by summing the abundance values of the peptides detected for each protein.

### Parallel Reaction Monitoring (PRM)-Targeted MS Analysis

Five microlitres of each sample (equivalent to 1 μg) was loaded online on a C18 PepMap 300 μm I.D. 0.3 × 5 mm trapping column (5 μm, 100 Å, Thermo Scientific) and analyzed by LC-ESI MSMS using a Thermo Ultimate 3000 RSLC nanoUPLC coupled to a Thermo Orbitrap Exploris OE240 mass spectrometer. Peptides were separated on a 15 cm, 75 μm ID column, with a flow rate of 250 nL/min and a 60-min long gradient. The liquid chromatographic system was coupled via a nanospray source to a mass spectrometer. The mass-spec method used worked in PRM mode, monitoring the selected peptides in light format. Selection and extraction of each of the transition areas (Supplementary Table 4) were carried out with the Skyline v21.2 software.^15^

### Functional Enrichment Analysis

Functional analysis of the differential proteins was done with Ingenuity Pathway Analysis (IPA) v.6875226 (Ingenuity Systems, www.ingenuity.com). The enrichment significance of the canonical pathways was calculated according to the following principles of the IPA user’s manual: (1) the ratio between the number of proteins from the experimental data set that map on a given pathway over the total number of proteins that belong to this pathway; and (2) Fisher’s exact test that was used to calculate a *p*-value associated with the probability that the association between the experimental set of proteins and the assigned pathway is not random. A pathway enrichment was considered statistically significant when *p* < 0.05. STRING v11.5 was also used to find protein interaction networks according to their functions.

### Statistical Analysis

Data are shown as the mean ± SEM. The number of replicates was indicated in the figure legends. Statistical differences were examined using the two-tailed Student’s *t*-test to compare two independent groups. Data from mass spectrometry analysis were processed using Proteome Discoverer 2.5 software. In all cases, a statistical significance of an adjusted *p*-value ≤ 0.05 was considered. Calculations and graphical representations were performed using GraphPad Prism version 9.4.1. software.

## Results and Discussion

**Figure 1:**
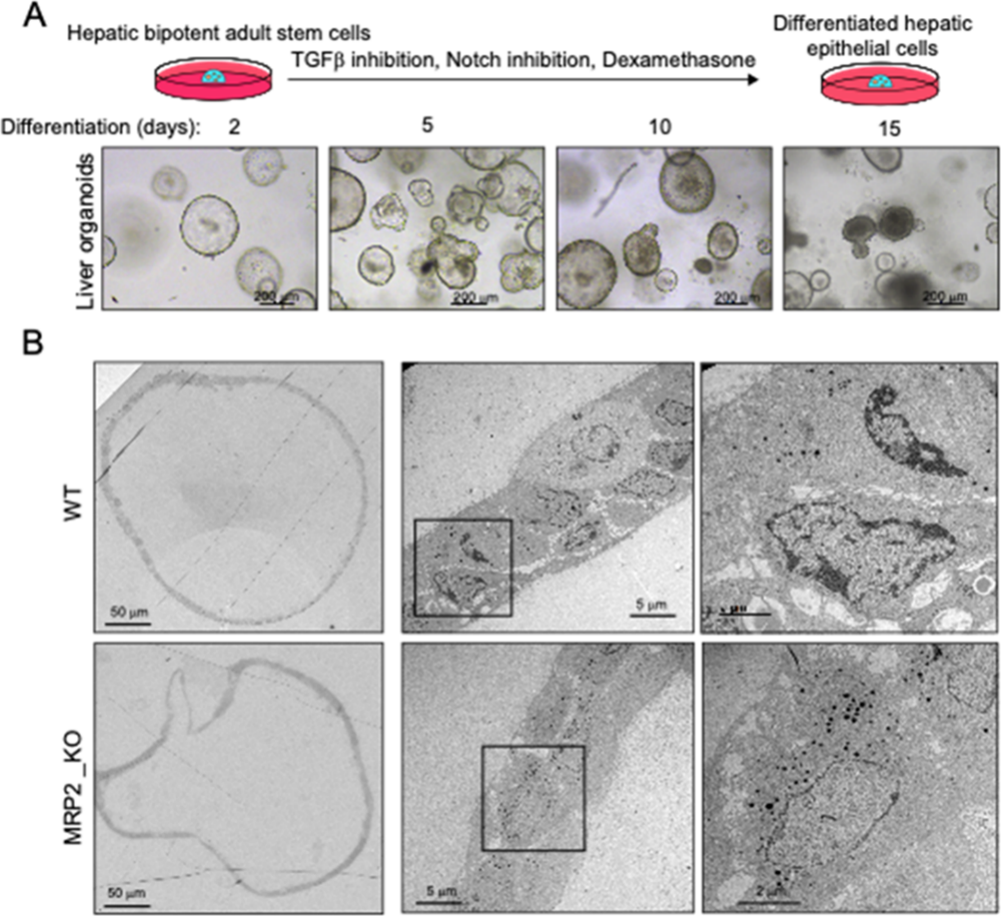
Electron microscopy analysis of MDR2KO mouse liver organoids. (A) Hepatic bipotent adult stem cells were isolated from hepatic tissue and placed in a 3D environment in the presence of expansion medium. (B) Differentiated organoids from WT and MDR2KO mice were fixed and processed for transmission electron microscopy. Cells are organized in sheets facing big central lumens (left and central images) and form smaller cavities between their lateral membranes containing cell–cell junctions and microvilli (right images), which have been shown to have functional features resembling bile canaliculi and contain canalicular markers.^11^

PFIC3 is a hereditary liver syndrome correlated with a severely compromised hepatic function and a challenging clinical management. Aiming to identify impaired hepatocyte processes associated with a deficiency of MDR2 before the onset of symptoms, we have done a systematic proteomic analysis of liver organoids from 3 month-old MDR2KO mice. Liver organoids were derived from expanding in 3D hepatic bipotent ductal stem cells obtained from wild type (WT) and MDR2KO mice. Once enough cellular material was obtained, cells were exposed to a differentiation medium containing dexamethasone and inhibitors of Notch and TGF-β for 15 days (Figure 1A). Differentiated organoids formed spheroids (Figure 1A,B). In addition to secreting albumin and processing CFDA (not shown),^9,11^ electron microscopy analyses confirmed that organoid cells formed lateral cavities between them, which have been shown to functionally resemble bile canaliculi upon differentiation.^11^

**Figure 2:**
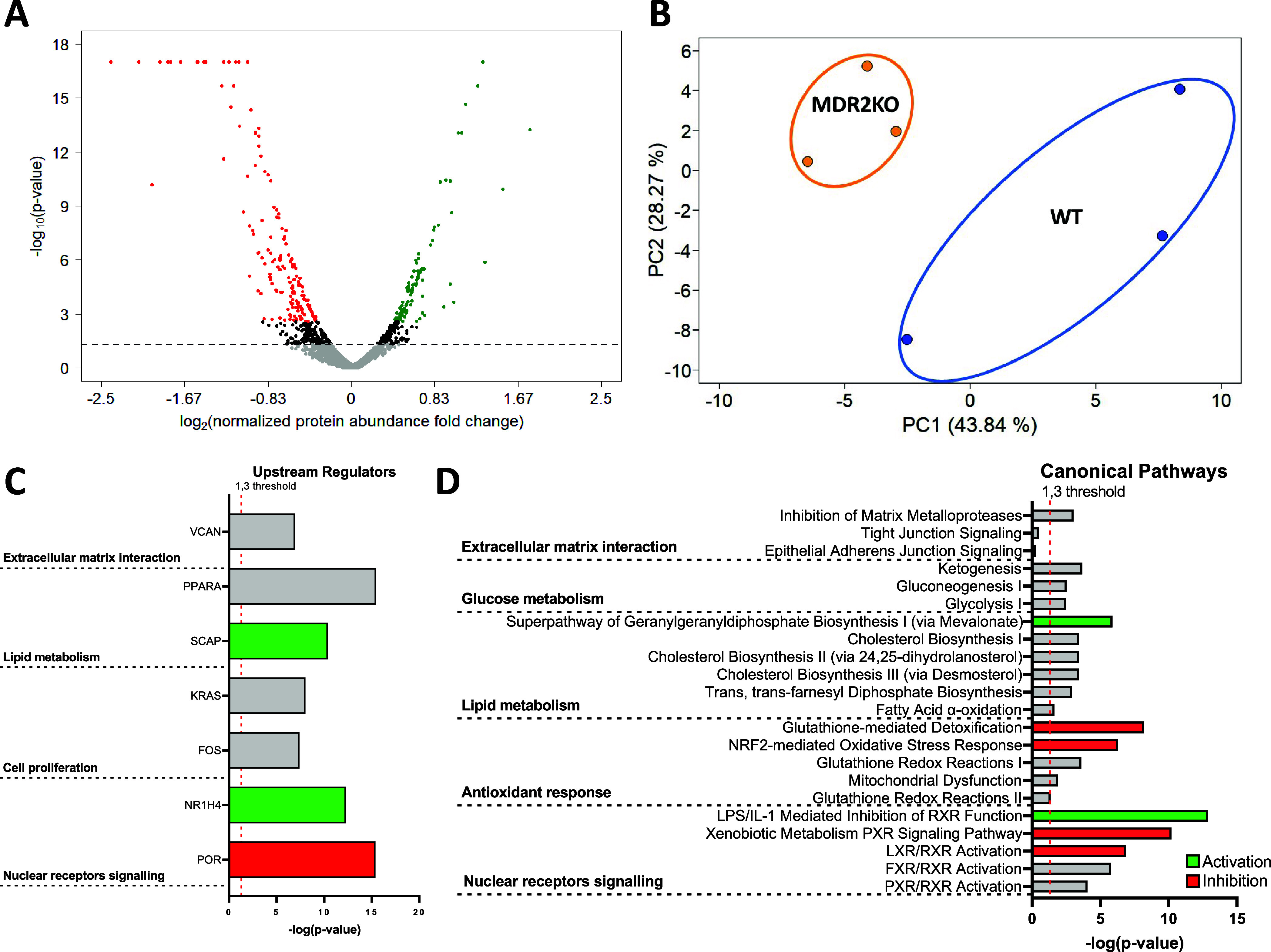
Shotgun proteomic analysis of MDR2KO mouse liver organoids. (A) Volcano plot showing the 174 downregulated (red) and 105 upregulated (green) proteins in MDR2KO organoids compared to WT organoids. (B) PCA plot displaying the segregation of WT and MDR2KO organoids. (C) Upstream regulators proposed by IPA as master drivers of the molecular phenotype of MDR2KO organoids based on the differential proteomics profile. (D) Statistically enriched canonical pathways suggested by IPA analysis based on the differential proteomics profile.

The efficiency of TMT labeling was over 91.5%, as determined with Proteome Discoverer. Using a cutoff FDR < 1%, we identified 54976 peptides corresponding to 5347 protein groups (Supplementary Table 5) of which 279 were differentially regulated (174 downregulated and 105 upregulated in MDR2KO/WT contrast) (Figure 2A and Supplementary Table 6). The PCA analysis showed good experimental reproducibility and sample clustering according to their corresponding biological condition (Figure 2B).

To investigate upstream checkpoints that would explain the differential protein profile in MDR2 KO organoids, we searched for master regulators. The top candidates were VCAN, PPARA, and KRAS (Figure 2C) which would coordinate the regulation of the cellular processes occurring in MDR2KO organoids, as suggested by IPA enrichment analysis: (1) Alterations of cell membrane composition and function, (2) Metabolic reprogramming, and (3) Cell proliferation and differentiation (Figure 2D). Changes on key representative proteins of the regulated cellular processes were further confirmed by PRM MS analysis (Supplementary Figure 1).

**Figure 3:**
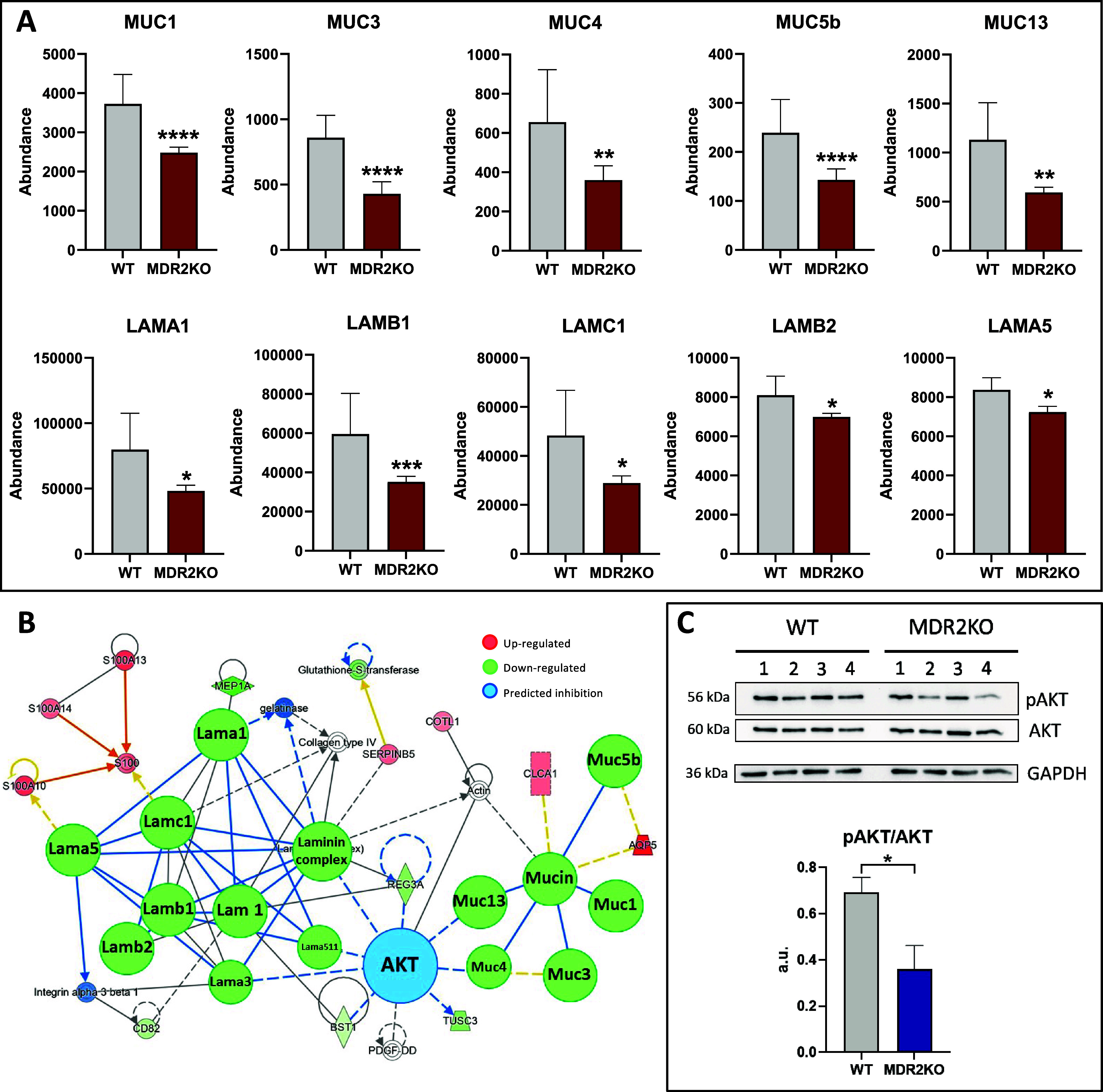
MDR2 deficiency alters the cell–ECM interaction. (A) Reduced abundance of mucins and laminins in MDR2KO organoids. Red: proteomic analysis. (B) Network representation (IPA) of the functional interaction between the differential proteins in MDR2KO organoids associated with cell–ECM interaction and the potential regulatory role of AKT (blue). (C) Western blot analysis showing the reduced phosphorylation of AKT in MDR2KO organoids (*n* = 4). Blue: Western blot. *p < 0.05, **p < 0.01, ***p < 0.005, ****p < 0.001.

The interaction between the ECM and cell membrane proteins modulates essential cellular processes, including cytoskeletal organization, cell growth, cell migration, and tissue development.^16^ In agreement with these observations, and despite the composition of the ECM being strongly influenced by the immunological environment that is not reproduced in the organoid system, we observed decreased levels of two main protein groups that participate in the ECM–cell communication, namely, mucins and laminins. MUC1, 3, 4, 5b, and 13 were downregulated in MDR2KO organoids (Figure 3A) which might indicate a severe condition at risk of progression to cancer. Indeed, mucins have been proposed as diagnostic and prognostic markers in HCC and cholangiocarcinome. Mucins are highly glycosylated, large, O-glycoproteins, which represent the major component of any mucous secretion. Their impaired regulation has been associated with a loss of epithelial cell polarity, progression to primary liver cancer, and epithelial–mesenchymal transition and metastases.^17^ Laminins belong to a family of heterotrimeric ECM glycoproteins, which play a major role in cell migration, differentiation, and tumor cell invasion. The communication of laminins with the cells is largely mediated by the interaction of the α-chain with integrin and nonintegrin receptors.^18^ Downregulation of LAMA1, LAMB1, LAMC1, LAMB2, and LAMA5 in MDR2KO compared with WT organoids (Figure 3A) suggests an impaired interaction of MDR2-deficient cells with the ECM and a potential alteration of cell growth, motility, and interaction. It is known that PI3K/AKT pathway is regulated by ECM interactors and cell adhesion proteins,^19^ in good agreement with our functional inferences based on the IPA enrichment analysis of the MDR2KO organoids differential proteins (Figure 3B). Moreover, PI3/AKT regulation was further confirmed by Western blot, which revealed a significant decrease of AKT phosphorylation in MDR2KO organoids and, therefore, the inhibition of this signaling pathway (Figure 3C).

**Figure 4:**
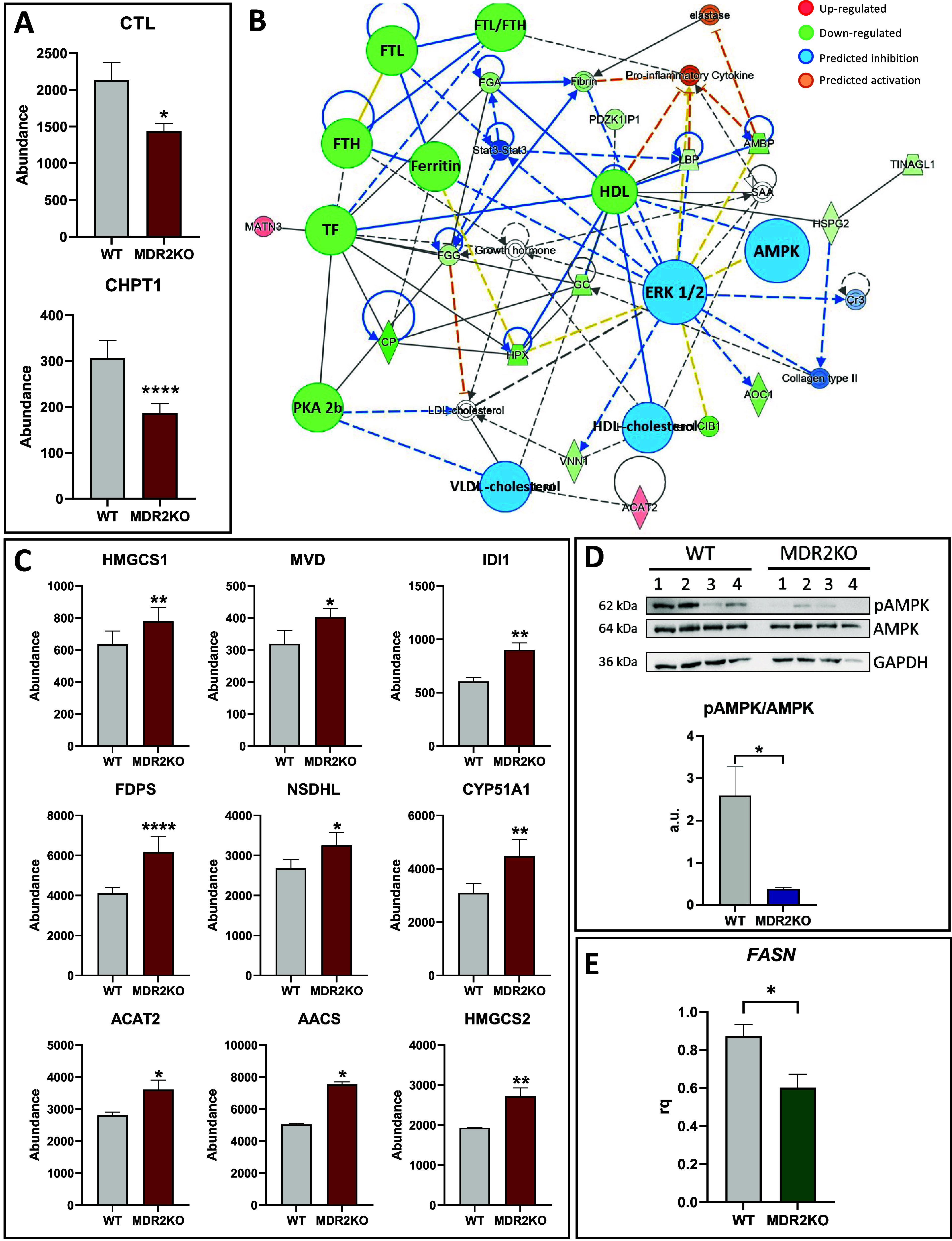
Regulation of lipid metabolism in MDR2KO organoids. (A) Downregulation of CTL and CHPT1 in MDR2KO organoids, indicating an impaired PC homeostasis. Red: proteomic analysis. (B) Functional network generated with IPA connecting the regulated proteins involved in molecular transport and cell maintenance in MDR2KO organoids. (C) Upregulation of enzymes involved in cholesterol synthesis in MDR2KO organoids. Red: proteomic analysis. (D) Western blot analysis showing the reduced phosphorylation of AMPK might explain the activation of HMGCR in MDR2KO organoids (*n* = 4). Blue: Western blot. (E) Downregulation of *FASN* suggesting downregulation of fatty acid synthesis in MDR2KO organoids. Green: RT-qPCR (*n* = 4). *p < 0.05, **p < 0.01.

MDR2 activity mediates the transport of PC across the basolateral membrane of hepatocytes^4^ therefore, we wondered if MDR2 deficiency might affect the synthesis of this phospholipid. Among the differentially expressed proteins, choline transporter like (CTL) and ChPT1 (cholinephosphotransferase 1), two essential proteins for the synthesis of PC, were significantly downregulated. Reduction of both, CTL, which transports choline inside the cell, and CHPT, which synthesizes PC from CDP-choline (Figure 4A)^20^ may suggest a mechanism triggered to compensate the lack of PC secretion that may impair the intracellular phospholipid balance and ultimately alter membrane composition and fluidity.^21^ In line with this hypothesis, molecular transport and cell maintenance pathways were among the regulated processes suggested by IPA analysis (Figure 4B). The inference was based on the repression of iron (FTH and FTL, ferritin heavy and light chain; TF, transferritin) and cholesterol (high-density lipoprotein, HDL and very-low-density lipoprotein, VLDL) transport. PC is the most abundant phospholipid in cells and is needed for HDL and VLDL assembly.^22^ Low PC availability would compromise lipoprotein production and normal hepatic cholesterol transport.

The differential protein profile described in this study also points to a significant reprogramming of lipid metabolism. Activation of cholesterol synthesis in MDR2-deficient organoids (Figure 4C) is supported by the upregulation of several enzymes catalyzing this process that include HMGCS1 (Hydroxymethylglutaryl-CoA synthase, cytoplasmic), MVD (Hydroxymethylglutaryl-CoA synthase), IDI1 (Isopentenyl-diphosphate Delta-isomerase 1), FDPS (Farnesyl pyrophosphate synthase), NSDHL (Sterol-4-alpha-carboxylate 3-dehydrogenase), CYP51A1 (Lanosterol 14-alpha demethylase), ACAT2 (Sterol O-acyltransferase 2), AACS (Acetoacetyl-CoA synthetase), HMGCS2 (Hydroxymethylglutaryl-CoA synthase, mitochondrial). HMGCR catalyzes the rate-limiting step of cholesterol synthesis and therefore is central to control cholesterol homeostasis. Although this protein has not been identified in our proteomics analysis, we found a significant reduction of AMPK phosphorylation at T172 under MDR2 deficiency (Figure 4D), suggesting its inactivation. Since HMGCR is negatively regulated by AMPK-mediated phosphorylation,^23^ it might be speculated that HMGCR activity is enhanced in MDR2KO organoids. It is worth to consider that a reduced phospholipid production concomitant to increased cholesterol levels may lead to cholesterol crystal formation that is one of the common symptoms of PFIC3 patients.^24^ Conversely to cholesterol, fatty acid synthesis appears to be downregulated in MDR2KO organoids, as evidenced by the significant downregulation of *FASN* expression (Figure 4E). Altogether these results suggest that MDR2 deficiency activates cholesterol production, perhaps coupled to energy production (Supplementary Figure 2).

**Figure 5:**
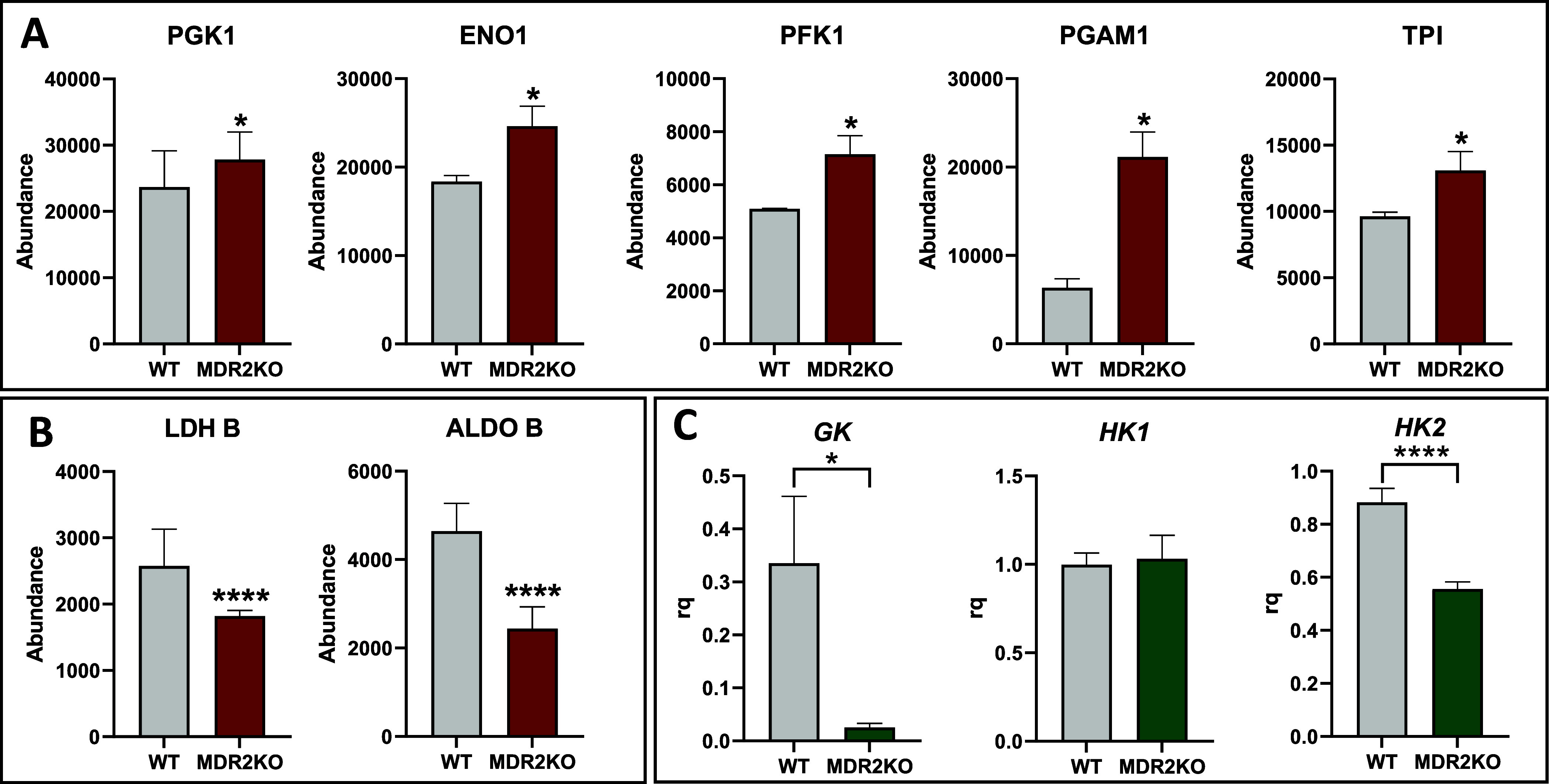
Rewiring of the intermediate metabolism in MDR2KO organoids. (A) Upregulation of glycolytic enzymes in MDR2KO organoids. Red: proteomic analysis. (B) Downregulation of gluconeogenic enzymes in MDR2KO organoids. (C) Downregulation of *GK* and *HK2* and maintenance of *HK1* levels in MDR2KO organoids. Green: RT-qPCR (*n* = 4). *p < 0.05, **p < 0.01.

Reduction of the AMPK catalytic activity also suggests regulation of the cellular energy metabolism in the MDR2KO organoids. AMPK is an energy sensor that activates or represses energy production in response to oscillations of intracellular ATP levels. This activity is performed by regulating the activity of metabolic enzymes either by controlling their phosphorylation or their abundance through modulation of transcription factors that regulate the expression of the corresponding coding genes.^25^ We observed a consistent upregulation of glycolytic proteins, including PGK (phosphoglycerate kinase); Eno1 (Alpha enolase); PFK1 (6-phosphofructo-2-kinase 1); PGAM1 (phosphoglycerate mutase 1); and TPI (triosephosphate isomerase) in MDR2KO organoids (Figure 5A). Inversely, other proteins such as the gluconeogenic protein ALDOB (aldolase B) and LDH B (lactate dehydrogenase b chain), which is involved in anaerobic glucose metabolism, were found to be downregulated in the MDR2KO group (Figure 5B). Previous studies have already demonstrated that ABCB4 is a modulator of glucose homeostasis in humans and mice through mechanisms that may involve LRH-1-dependent PC pathways and that ABCB4 deficiency improves glucose tolerance.^26^ These results indicate that MDR2KO organoid cells stimulate energy production by consuming glucose to synthesize ATP via the aerobic pathway.

In order to further investigate the reprogramming of glucose metabolism in MDR2KO organoids, we next measured the expression levels of hexokinase (*HK*), which catalyzes the first and one of the rate-limiting steps of glycolysis, where glucose is phosphorylated to glucose-6-phosphate. Of the three measured isoforms, *HK1* showed no differences while the glucokinase (*GK*) and *HK2* expression were significantly decreased in MDR2KO than in the WT group. (Figure 5C). GK is the hepatocyte-specific isoform;^27^ therefore, its reduction suggests that the loss of MDR2 might compromise the differentiated and metabolic phenotype of hepatocytes. *HK2* has been associated with tumor progression, which is characterized by active proliferation,^28^ so its downregulation, may suggest an impairment of the proliferative capacity of MDR2-deficient organoids, in agreement with the aforementioned inhibition of the EGFR/ERK pathway as discussed below. Finally, we investigated the regulation of gluconeogenesis by analyzing the mRNA levels of two FOXO1 target genes. FOXO1 is an AMPK-regulated factor that controls the transcription of genes encoding gluconeogenic enzymes, such as *PEPCK* and *G6 Pase*. Both, *PEPCK* and G6 Pase mRNA levels were unaffected by MDR2 deficiency (Supplementary Figure 3). These results are in line with AMPK inactivation and support the reorganization of cellular metabolism toward the consumption of glucose for ATP synthesis in MDR2KO organoids.

**Figure 6:**
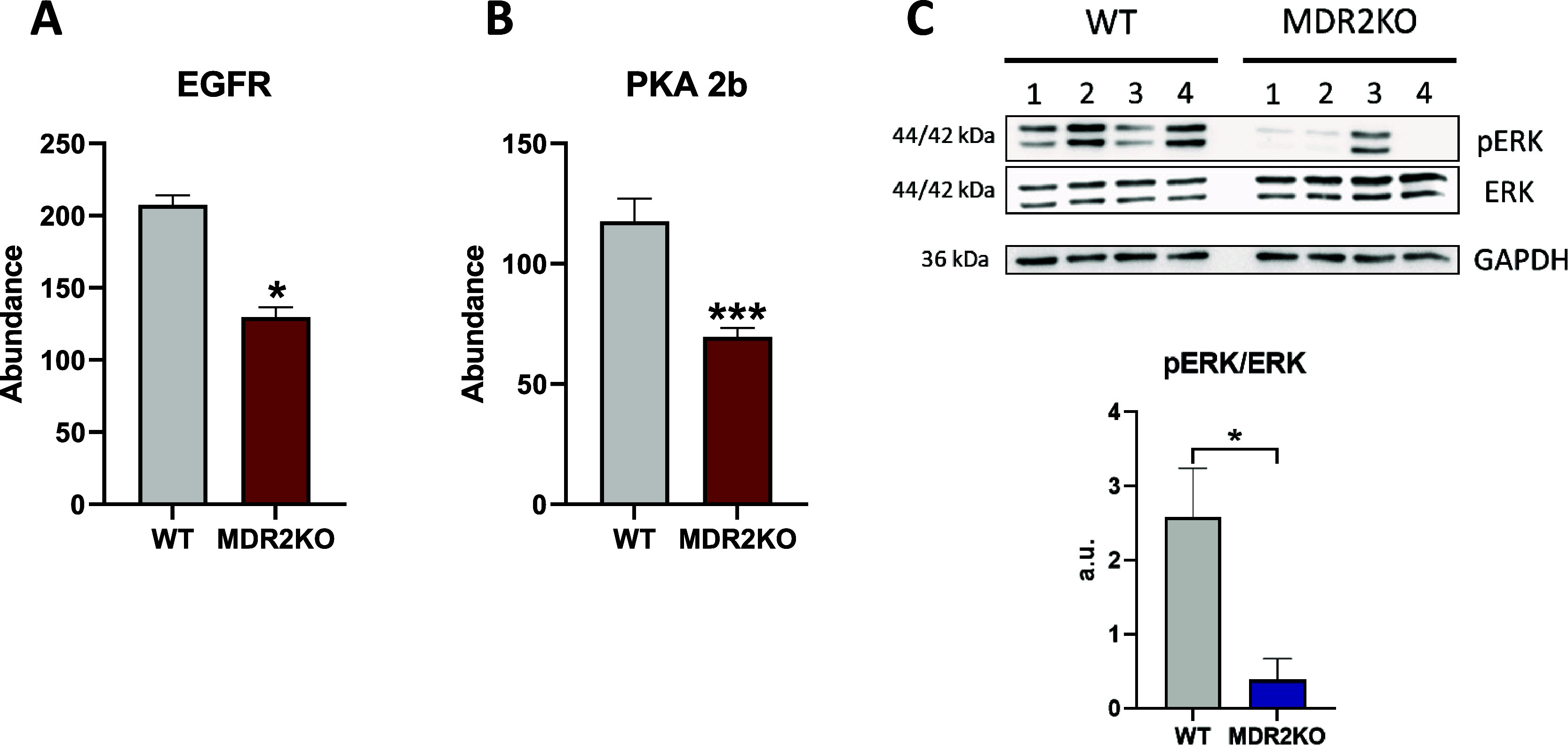
MDR2 deficiency regulates cell proliferation and differentiation. (A) Downregulation of EGFR in MDR2KO organoids. Red: proteomic analysis. (B) Downregulation of PKA2b in MDR2KO organoids. Red: proteomic analysis. (C) Western blot showing the reduced phosphorylation of ERK in MDR2KO organoids, (*n* = 4). Blue: Western blot. *p < 0.05, ***p < 0.005.

The functional enrichment analysis points toward EGFR downregulation in MDR2-deficient organoids. EGFR is a pivotal regulator of liver cell fate controlling essential functions as liver regeneration and response to liver damage^29^ and therefore its deregulation might be one of the factors driving the progression of PFIC3 (Figure 6A). EGFR decrease parallels PKA 2b drop, providing a mechanistic support to EGFR impairment, since it has been shown that PKA inhibition induces EGFR endocytosis and degradation in the proteasome^30^ (Figure 6B). In line with these observations is also the aforementioned AKT hypophosphorylation as EGFR is one of the upstream activators of the PI3K/AKT pathway. Similarly, RAS is phosphorylated by the EGFR kinase activity, leading to activation of its downstream pathway. EGFR decrease would impair RAS activation, in agreement with the prediction of downstream ERK inhibition in our functional analysis, which was further confirmed by Western blot (Figure 6C). These results reveal EGFR as a master regulator of the signaling rewiring induced by MDR2 deficiency (Supplementary Figure 4) and are coincident with recent observations indicating that the absence of EGFR tyrosine kinase activity in albumin-expressing cells leads to reduced and delayed liver damage and more efficient regeneration upon a cholestatic injury, concomitantly with a shift from a profibrotic to a restorative inflammatory response and an enhanced expansion of oval cells.^31^

Finally, the impairment of liver-specific functions, such as xenobiotic metabolism, VLDL and HDL production, and the reduced GK expression, suggests poor cell differentiation of MDR2-deficient organoids. Taking together, these observations suggest that MDR2 lack impairs hepatocyte differentiation, resulting either from a deleterious effect in the deficient organoid biology or, alternatively, from an interference with the cell differentiation process induced during the obtention of the organoids. Further investigation would provide additional data to answer this question.

In summary, 3D cellular models provide an excellent framework to dissect the molecular background of biological/pathological phenotypes. The lack of MDR2 induces significant proteome alterations in liver organoids notwithstanding signals from other organs and tissues. Changes in cell membrane and ECM composition, as well as a rewiring of energy metabolism, are major components of MDR2KO organoids. EGFR downregulation emerges as a main driver of these alterations. These results fit well with the molecular landscape recently reported in the liver of PFIC3 patients,^10^ suggesting that the described proteomic remodeling might provide relevant insights on the early events associated with the progression of the disease.

## Supporting Information Available

Primers used for RT-qPCR; antibodies used for Western blot; TMT-6plex organoid samples labeling; peptides used for PRM assays; protein groups identified in proteomic analysis; differential proteins in proteomic analysis; PRM MS analysis; schematic cholesterol synthesis pathway; mRNA levels of FOXO1 target genes; EGFR/PKA/ERK schematic pathway; and Western blot entire membranes (PDF)
Protein groups (XLSX)
