# Supplementary material for: Molecular Insights of Cholestasis in MDR2 Knockout Murine Liver Organoids
Source: J Proteome Res. 2024 Mar 15;23(4):1433–42. doi: 10.1021/acs.jproteome.3c00900 (PMC11002922; doi:10.1021/acs.jproteome.3c00900)

Molecular insights of cholestasis in MDR2 knockout murine liver organoids.

Irene Blázquez-García[1], Laura Guerrero[1], Cristina Cacho-Navas[2], Nabil Djouder[3], Jaime Millan[2], Alberto Paradela[1], Lorena Carmona-Rodríguez[1#] and Fernando J. Corrales[1#*]

[1] Functional Proteomics Laboratory, Centro Nacional de Biotecnología (CSIC), Madrid 28049, Spain.

[2] Centro de Biología Molecular Severo Ochoa (CBMSO), Madrid 28049, Spain.

[3] Centro Nacional de Investigaciones Oncológicas (CNIO), Madrid 28029, Spain

[#] These authors share senior authorship

* To whom correspondence should be addressed

Corresponding Author

Fernando J. Corrales, Ph.D.

Functional Proteomics Laboratory

National Center for Biotechnology, CNB-CSIC

Darwin, 3

28049 Madrid

Spain

Tlf: +34 915854694

Email: fcorrales@cnb.csic.es

Lorena Carmona-Rodríguez (ORCID ID: 0000-0001-5937-5577)

Fernando Corrales (ORCID ID: 0000-0002-0231-5159)

SUPPLEMENTARY MATERIAL

Supplementary table 1. Primers used for RT-qPCR.

Gen	Gene ID	Description	Forward primer (5' → 3')	Reverse primer (5' → 3')
FASN	14104	Fatty acid synthase	CACAGTGCTCAAAGGACATGCC	CACCAGGTGTAGTGCCTTCCTC
GCK	103988	Glucokinase	GCATCTCTGACTTCCTGGACAAG	CTTGGTCCAGTTGAGCAGGATG
HK1	15275	Hexokinase-1	GAAAGGAGACCAACAGCAGAGC	TTCGTTCCTCCGAGATCCAAGG
HK2	15277	Hexokinase-2	CCCTGTGAAGATGTTGCCCACT	CCTTCGCTTGCCATTACGCACG
GAPDH	14433	Glyceraldehyde-3-phosphate dehydrogenase	CAATGAATACGGCTACAGCAAC	AGGGAGATGCTCAGTGTTGG

Supplementary table 2. Antibodies used for Western blot.

Antibodies	Dilution	Host	Ref	Company
Anti-phospho AMPK	1:10000	Rabbit	#2535	Cell Signaling
Anti-AMPK	1:500	Mouse	ab110036	Abcam
Anti-phospho AKT	1:1000	Rabbit	ab192623	Abcam
Anti-AKT	1:2000	Mouse	#2920	Cell Signaling
Anti-phospho ERK	1:1000	Rabbit	#4370	Cell Signaling
Anti-ERK	1:1000	Rabbit	#4695	Cell Signaling
Anti-GAPDH	1:2000	Mouse	ab8245	Abcam
Goat Anti-Rabbit IgG (HRP)	1:2000	Goat	P0448	DAKO
Goat Anti-Mouse IgG (HRP)	1:2000	Goat	P0447	DAKO

Supplementary table 3. TMT-6plex organoid samples labeling.

TMT-6plex Tag	126	127	128	129	130	131
Sample	WT-162	MDR2KO-160	WT-166	MDR2KO-161	WT-170	MDR2KO-166

Supplementary Table 4. Peptides used in PRM assays

Protein ID	*Peptide Sequence*	*Precursor Mz*	*Precursor Charge*	*Transition m/z*	*Transition*
Q61001	CDVGGALGQGCEPK	724,318851	2	1073,50442	y11
				888,424378	y8
				775,340314	y7
				244,165568	y2
				276,064868	b2
	AGALLPAIR	441,281994	2	753,498135	y7
				682,461021	y6
				569,376957	y5
				456,292893	y4
				200,102967	b3
	ALFSQTSSSVSLR	691,867348	2	1051,537828	y10
				836,447222	y8
				735,399543	y7
				648,367515	y6
				185,128454	b2
	DLGAQGAVAEAELAEAQR	899,950129	2	1087,537828	y10
				1016,500714	y9
				887,458121	y8
				816,421007	y7
				286,139747	b3
	GQLQLVEGNFR	630,838393	2	834,446828	y7
				721,362764	y6
				622,29435	y5
				493,251757	y4
				186,087317	b2
P19467	TDYGQTVIIK	569,311146	2	921,540394	y8
				758,477066	y7
				260,196868	y2
				217,081898	b2
	CQDILQCTCKPGLDR	621,955184	3	1106,508125	y9
				946,477476	y8
				845,429798	y7
				557,304186	y5
				404,123445	b3
	LIEDDFHNLR	424,550809	3	916,427155	y7
				801,400212	y6
				686,373269	y5
				539,304855	y4
				402,245943	y3
E9PWP7	YTPGFENTLDTVVK	792,40122	2	1018,541516	y9
				889,498923	y8
				775,455996	y7
				446,29731	y4
				246,181218	y2
	IQIQRPQVVMTSL	764,929425	2	1287,708933	y11
				566,285425	y5
				242,149918	b2
	VQNSATVSVNPEETCK	881,91744	2	877,372008	y7

				763,329081	y6
				537,233724	y4
				408,191131	y3
				228,134267	b2
Q91VA1	FLLPSTPALGR	586,345323	2	911,530892	y9
				798,446828	y8
				701,394064	y7
				513,314357	y5
				261,159754	b2
	VILEYIDHK	377,213246	3	804,388644	y6
				675,346051	y5
				399,198659	y3
				284,171716	y2
Q920E5	ELGHPEIGDAIAR	459,908885	3	602,32565	y6
				430,277243	y4
				359,24013	y3
				246,156066	y2
	EVLEYNALGGK	596,814053	2	964,509822	y9
				851,425758	y8
				722,383165	y7
				261,155731	y3
				229,118283	b2
	GLTVVQAFQELVEPK	829,461612	2	1060,567337	y9
				989,530223	y8
				244,165568	y2
				171,112804	b2
				272,160482	b3
	QILEENYGQK	611,309134	2	980,468351	y8
				867,384287	y7
				738,341694	y6
				332,192845	y3
Q8K0C4	EYFQSWGESGER	737,815312	2	907,390435	y8
				634,279094	y6
				577,25763	y5
				448,215037	y4
				293,113198	b2
	NEDLNAEEVYGR	704,820595	2	937,437386	y8
				823,394458	y7
				752,357344	y6
				244,092797	b2
				359,11974	b3
Q8JZK9	LEDTYFDR	529,743096	2	945,394852	y7
				816,352259	y6
				701,325316	y5
				600,277637	y4
	NSIYSGLEAFGDVK	750,372463	2	1022,515302	y10
				636,335152	y6
				565,298038	y5
				202,082232	b2
				315,166296	b3
	TCVAPDVFAENMK	749,339252	2	755,339252	y6

				608,270838	y5
				262,085603	b2
				361,154017	b3
Q91Y97	ALQASALAAWGGK	622,343312	2	931,499592	y10
				589,309272	y6
				518,272158	y5
				447,235044	y4
	ETTIQGLDGLSER	709,85972	2	974,490149	y9
				846,431572	y8
				789,410108	y7
				676,326044	y6
				561,299101	y5
	GILAADESVGTMGNR	753,864479	2	837,388327	y8
				773,390042	y8 -64
				651,287885	y6
				284,196868	b3
Q01279	IPLENLQIIR	604,871705	2	998,599306	y8
				885,515242	y7
				756,472649	y6
				529,345657	y4
				211,144104	b2
	NLQEILIGAVR	613,366787	2	870,540728	y8
				741,498135	y7
				515,330007	y5
				402,245943	y4
				356,192845	b3
P31324	EGEHVIDQGDDGDNFYVIDR	765,004971	3	665,361701	y5
				502,298373	y4
				403,229959	y3
				552,241252	b5
	AATITATSPGALWGLDR	850,951943	2	1344,69064	y13
				1243,642962	y12
				1172,605848	y11
				1071,558169	y10
				984,526141	y9
				646,330736	y5
				244,129182	b3

Supplementary table 6. Differential proteins in proteomic analysis.

Protein ID	Protein Name	Gene Symbol	Log2(ratio)	Adj. P-Value
**Q03404**	Trefoil factor 2	Tff2	1,78	1,1308E-11
**P53996-1**	Cellular nucleic acid-binding protein	Cnbp	1,51	1,4831E-08
**Q80V70**	Multiple epidermal growth factor-like domains protein 6	Megf6	1,33	8,0446E-05
**O88312**	Anterior gradient protein 2 homolog	Agr2	1,31	3,0506E-15
**Q9D2J4**	V-set and immunoglobulin domain-containing protein 1	Vsig1	1,26	5,7316E-14
**Q02013**	aquaporin-1	Aqp1	1,14	5,452E-13
**Q9WTY4**	Aquaporin-5	Aqp5	1,1	1,7262E-11
**Q05816**	Fatty acid-binding protein, epidermal	Fabp5	1,07	1,6238E-11
**Q8VD31**	Tapasin-related protein	Tapbpl	1,02	0,00688517
**Q8K1A5**	Transmembrane protein 41B	Tmem41b	1	2,5469E-07
**P06795**	multidrug resistance protein 1B	Abcb1b	0,99	5,7633E-09
**P70269**	Cathepsin E	Ctse	0,99	0,00095553
**Q8BHB9**	Chloride intracellular channel protein 6	Clic6	0,99	5,6025E-09
**P99027**	60S acidic ribosomal protein P2	Rplp2	0,94	5,194E-09
**Q61062**	segment polarity protein dishevelled homolog DVL-3	Dvl3	0,92	0,01138439
**P47739**	Aldehyde dehydrogenase, dimeric NADP-preferring	Aldh3a1	0,89	6,3947E-09
**P54227**	Stathmin	Stmn1	0,87	1,1617E-06
**Q920E5**	Farnesyl pyrophosphate synthase	Fdps	0,83	1,542E-06
**P31786**	acyl-CoA-binding protein	Dbi	0,82	1,9887E-06
**Q8VHB5**	Carbonic anhydrase 9	Ca9	0,81	7,1015E-06
**Q6ZWY8**	thymosin beta-10	Tmsb10	0,79	1,2963E-05
**Q99N50-1**	Synaptotagmin-like protein 2	Sytl2	0,73	0,02607295
**P13011**	Acyl-CoA desaturase 2	Scd2	0,72	0,00016788
**Q9D168**	integrator complex subunit 12	Ints12	0,71	0,02086927
**Q9D311**	Dual oxidase maturation factor 2	Duoxa2	0,71	0,00358957
**Q9WTW2**	potassium voltage-gated channel subfamily E member 3	Kcne3	0,71	0,00063053
**P02798**	metallothionein-2	Mt2	0,7	0,00016788
**Q6PGH2**	Jupiter microtubule associated homolog 2	Jpt2	0,69	0,00019492
**Q80SZ7**	guanine nucleotide-binding protein g(i)/g(s)/g(o) subunit gamma-5	Gng5	0,69	0,00025934
**Q9EQU5-2**	Isoform 2 of Protein SET	Set	0,68	0,03619435
**P08207**	Protein S100-A10	S100a10	0,67	5,6304E-05
**Q8R3P0**	aspartoacylase	Aspa	0,67	0,00031534
**Q9D939**	Sulfotransferase 1C2	Sult1c2	0,67	3,1701E-05
**A2ARZ3**	fibrous sheath-interacting protein 2	Fsip2	0,66	0,00049617
**P14069**	protein S100-A6	S100a6	0,66	0,00022315
**Q9D7Z6**	Calcium-activated chloride channel regulator 1	Clca1	0,66	0,00041857
**P25085**	Interleukin-1 receptor antagonist protein	Il1rn	0,65	0,00060683
**Q61166**	Microtubule-associated protein RP/EB family member 1	Mapre1	0,65	9,0994E-05
**Q7TNS2**	MICOS complex subunit Mic10	Micos10	0,65	0,00089529
**Q8VE95**	UPF0598 protein C8orf82 homolog	-	0,65	0,04778423
**Q9CY34**	NEDD8-conjugating enzyme UBE2F	Ube2f	0,65	0,00063053
**P05063**	Fructose-bisphosphate aldolase C	Aldoc	0,64	0,00104835

**Q8R1L4**	ER lumen protein-retaining receptor 3	Kdelr3	0,64	6,7774E-05
**Q9JHJ0**	tropomodulin-3	Tmod3	0,63	0,00098278
**Q8R0Y6**	Cytosolic 10-formyltetrahydrofolate dehydrogenase	Aldh1l1	0,62	0,00021663
**P58044**	Isopentenyl-diphosphate Delta-isomerase 1	Idi1	0,61	0,00193147
**Q0VFX2**	Cilia- and flagella-associated protein 157	Cfap157	0,61	0,00177757
**Q8C142**	Low density lipoprotein receptor adapter protein 1	Ldlrap1	0,61	0,00154155
**Q8JZK9**	hydroxymethylglutaryl-CoA synthase, cytoplasmic	Hmgcs1	0,61	0,00105977
**O70456**	14-3-3 protein sigma	Sfn	0,6	0,0059865
**Q9CWF2**	Tubulin beta-2B chain	Tubb2b	0,6	0,00041857
**Q8K0C4**	lanosterol 14-alpha demethylase	Cyp51a1	0,59	0,00188233
**Q9CQI6**	coactosin-like protein	Cotl1	0,59	0,00293026
**Q9D1H8**	39S ribosomal protein L53, mitochondrial	Mrpl53	0,59	0,00910671
**P14206**	40S ribosomal protein SA	Rpsa	0,57	0,00155984
**P15105**	Glutamine synthetase	Glul	0,57	0,00124664
**P43275**	Histone H1.1	H1-1	0,57	0,00086295
**O35640**	Annexin A8	Anxa8	0,56	0,00208759
**P54869**	Hydroxymethylglutaryl-CoA synthase, mitochondrial	Hmgcs2	0,56	0,00184707
**P56382**	ATP synthase subunit epsilon, mitochondrial	Atp5f1e	0,56	0,00643207
**P97352**	Protein S100-A13	S100a13	0,56	0,00170077
**Q99K85**	phosphoserine aminotransferase	Psat1	0,56	0,0063433
**A2AQ99**	Dual oxidase 2	Duox2	0,55	0,00140492
**E9Q616**	AHNAK nucleoprotein (desmoyokin)	Ahnak	0,55	0,0171025
**Q8R3L5-1**	solute carrier organic anion transporter family member 3A1	Slco3a1	0,55	0,00854233
**O09117-1**	Synaptophysin-like protein 1	Sypl1	0,54	0,02104994
**P09411**	phosphoglycerate kinase 1	Pgk1	0,54	0,01025187
**Q5U430**	E3 ubiquitin-protein ligase UBR3	Ubr3	0,53	0,03060033
**Q8BLX4**	GDP-fucose transporter 1	Slc35c1	0,53	0,01361121
**Q9D2R0**	acetoacetyl-CoA synthetase	Aacs	0,53	0,01470488
**O35955**	Proteasome subunit beta type-10	Psmb10	0,52	0,01407875
**P24472**	Glutathione S-transferase A4	Gsta4	0,52	0,01506039
**P57787**	Monocarboxylate transporter 4	Slc16a3	0,52	0,01722165
**Q8JZN7**	Mitochondrial Rho GTPase 2	Rhot2	0,51	0,04749974
**Q9CQE8**	UPF0568 protein C14orf166 homolog	RTRAF	0,51	0,00679332
**Q9D2Q8**	protein S100-A14	S100a14	0,51	0,01636631
**P12382**	ATP-dependent 6-phosphofructokinase, liver type	Pfkl	0,5	0,02747511
**Q61881**	DNA replication licensing factor MCM7	Mcm7	0,5	0,02698693
**Q6IRU5-2**	Isoform 2 of Clathrin light chain B	Cltb	0,5	0,01015087
**Q9D5V6**	Synapse-associated protein 1	Syap1	0,5	0,01722165
**Q9D832**	DnaJ homolog subfamily B member 4	Dnajb4	0,5	0,02494318
**Q9D8Y0**	EF-hand domain-containing protein D2	Efhd2	0,5	0,01615864
**Q9DBJ1**	Phosphoglycerate mutase 1	Pgam1	0,5	0,02347545
**Q60716**	Prolyl 4-hydroxylase subunit alpha-2	P4ha2	0,49	0,02126025
**Q8BGV0**	probable asparagine--tRNA ligase, mitochondrial	Nars2	0,49	0,01252402
**Q99JF5**	Diphosphomevalonate decarboxylase	Mvd	0,49	0,02763231
**P17751**	Triosephosphate isomerase		0,48	0,02666257
**P18828**	syndecan-1	Sdc1	0,48	0,03903022
**Q00915**	Retinol-binding protein 1	Rbp1	0,48	0,02419914
**Q64442**	Sorbitol dehydrogenase	Sord	0,48	0,03577287

**Q64518**	Sarcoplasmic/endoplasmic reticulum calcium atpase 3		0,48	0,01268976
**P17182**	alpha-enolase	Eno1	0,47	0,04929389
**P70124**	Serpin B5	Serpinb5	0,47	0,04225678
**P97823-1**	Acyl-protein thioesterase 1	Lypla1; LYPLA1	0,46	0,05153705
**Q60854**	serpin B6	Serpinb6	0,46	0,05372408
**Q8CAY6**	Acetyl-CoA acetyltransferase, cytosolic	Acat2	0,46	0,03451258
**Q9CQQ8**	U6 snRNA-associated Sm-like protein LSm7	Lsm7	0,46	0,04469716
**P26350**	Prothymosin alpha	Ptma	0,45	0,02035755
**P53996-3**	Isoform 3 of Cellular nucleic acid-binding protein	Cnbp	0,44	0,03703411
**P61087**	Ubiquitin-conjugating enzyme E2 K	Ube2k	0,44	0,03386372
**Q80XU3**	Nuclear ubiquitous casein and cyclin-dependent kinase substrate 1	Nucks1	0,44	0,05172779
**Q9R1J0**	sterol-4-alpha-carboxylate 3-dehydrogenase, decarboxylating	Nsdhl	0,44	0,04551844
**P48997**	Involucrin	Ivl	0,43	0,05429474
**Q3UZ39-1**	Leucine-rich repeat flightless-interacting protein 1	Lrrfip1	0,43	0,05463207
**Q9D172**	ES1 protein homolog, mitochondrial	Gatd3a	0,43	0,03919054
**P02468**	Laminin subunit gamma-1	Lamc1	-0,35	0,05085424
**P19137**	Laminin subunit alpha-1	-	-0,36	0,02953045
**Q61001**	Laminin subunit alpha-5	Lama5	-0,36	0,04237028
**Q61805**	lipopolysaccharide-binding protein	Lbp	-0,36	0,04296721
**Q99JR5**	Tubulointerstitial nephritis antigen-like	Tinagl1	-0,36	0,04513711
**P11672**	Neutrophil gelatinase-associated lipocalin	Lcn2	-0,37	0,0331769
**P12023**	Amyloid-beta A4 protein	App	-0,37	0,03873031
**Q91VA1**	choline transporter-like protein 4	Slc44a4	-0,37	0,03563092
**O88593**	peptidoglycan recognition protein 1	Pglyrp1	-0,38	0,02943763
**P13707**	Glycerol-3-phosphate dehydrogenase [NAD(+)], cytoplasmic	Gpd1	-0,39	0,02226948
**P49935**	Pro-cathepsin H	Ctsh	-0,39	0,02283977
**P99029**	Peroxiredoxin-5, mitochondrial	Prdx5	-0,39	0,02645676
**Q9CYH2**	Redox-regulatory protein FAM213A	Prxl2a	-0,39	0,02664706
**Q9WUL7**	ADP-ribosylation factor-like protein 3	Arl3	-0,39	0,02666257
**P07744**	Keratin, type II cytoskeletal 4	Krt4	-0,4	0,05408419
**P10639**	thioredoxin	Txn	-0,4	0,01956735
**P48774**	Glutathione S-transferase Mu 5	Gstm5	-0,41	0,0154892
**Q61292**	laminin subunit beta-2	Lamb2	-0,41	0,01362575
**P21956-1**	Lactadherin	Mfge8	-0,42	0,01194246
**P52760**	2-iminobutanoate/2-iminopropanoate deaminase	Rida	-0,42	0,01362575
**Q08509**	Epidermal growth factor receptor kinase substrate 8	Eps8	-0,42	0,01037756
**Q8BH86**	D-glutamate cyclase, mitochondrial	Dglucy	-0,43	0,0106405
**P70665**	sialate O-acetylesterase	Siae	-0,44	0,04749974
**Q05793**	Basement membrane-specific heparan sulfate proteoglycan core protein	Hspg2	-0,44	0,00923641
**Q9Z0K8**	Pantetheinase	Vnn1	-0,44	0,00572019
**Q64462**	Cytochrome P450 4B1	Cyp4b1	-0,46	0,04740968
**Q78IQ7**	Zinc transporter ZIP4	Slc39a4	-0,46	0,00577519
**Q8BXZ1**	Protein disulfide-isomerase TMX3	Tmx3	-0,47	0,02486988
**Q61129**	Complement factor I	Cfi	-0,48	0,00473453
**Q99PS0**	Keratin, type I cytoskeletal 23	Krt23	-0,48	0,00338165
**P08122**	Collagen alpha-2(IV) chain	Col4a2	-0,49	0,00217697

**P45700**	Mannosyl-oligosaccharide 1,2-alpha-mannosidase IA	Man1a1	-0,49	0,01407875
**Q62141**	paired amphipathic helix protein sin3b	Sin3b	-0,49	0,00127241
**Q9DAW9**	Calponin-3	Cnn3	-0,49	0,01431303
**Q9QWR8**	alpha-N-acetylgalactosaminidase	Naga	-0,49	0,02091596
**Q571E4**	N-acetylgalactosamine-6-sulfatase	Galns	-0,5	0,01362575
**Q8BI08**	protein MAL2	Mal2	-0,5	0,00326442
**Q8K0E8**	Fibrinogen beta chain	Fgb	-0,5	0,03862916
**Q8K4X7**	1-acyl-sn-glycerol-3-phosphate acyltransferase delta	Agpat4	-0,5	0,01505222
**O88848**	ADP-ribosylation factor-like protein 6	Arl6	-0,51	0,03785153
**Q32NZ6-1**	Transmembrane channel-like protein 5	Tmc5	-0,51	0,01146308
**Q9D816**	Cytochrome P450 2C55	Cyp2c55	-0,51	0,03875644
**E9PV24**	Fibrinogen alpha chain	Fga	-0,52	0,0004833
**P01027-1**	Complement C3	C3	-0,52	0,00100335
**Q8K009**	Mitochondrial 10-formyltetrahydrofolate dehydrogenase	Aldh1l2	-0,52	0,02856879
**P02802**	Metallothionein-1	Mt1	-0,53	0,00070316
**P06909**	complement factor H	Cfh	-0,53	0,00105977
**P41216**	Long-chain-fatty-acid--CoA ligase 1	Acsl1	-0,53	0,00077108
**P47791-2**	Isoform Cytoplasmic of Glutathione reductase, mitochondrial	Gsr	-0,53	0,01007465
**Q8CCK0**	Core histone macro-H2A.2	Macroh2a2	-0,53	0,00764204
**P19467**	Mucin-13	Muc13	-0,54	0,01256995
**Q8BTY8-1**	Sec1 family domain-containing protein 2	Scfd2	-0,54	0,0171025
**Q99JF8**	PC4 and SFRS1-interacting protein	Psip1	-0,54	0,00889095
**Q9JM62-1**	Receptor expression-enhancing protein 6	Reep6	-0,54	0,00727551
**Q3UZZ6**	Sulfotransferase 1 family member D1	Sult1d1	-0,55	0,00039455
**Q8K0S2**	Glutamate-rich protein 5	Erich5	-0,55	0,03836257
**Q91WG0**	Acylcarnitine hydrolase	Ces2c	-0,55	0,0058441
**Q9ES64**	Harmonin	Ush1c	-0,55	0,04234294
**O54879**	High mobility group protein B3	Hmgb3	-0,56	0,00016461
**P70158**	acid sphingomyelinase-like phosphodiesterase 3a	Smpdl3a	-0,56	0,05006929
**Q01279**	epidermal growth factor receptor	Egfr	-0,56	0,01194246
**P19324**	Serpin H1	Serpinh1	-0,57	0,00060424
**Q64133**	Amine oxidase [flavin-containing] A	Maoa	-0,57	0,00012795
**Q8VCM7**	Fibrinogen gamma chain	Fgg	-0,57	0,02939013
**Q9JHF5**	V-type proton ATPase subunit a	Tcirg1	-0,57	0,00242491
**Q3UW68**	Calpain-13	Capn13	-0,58	0,00208759
**Q61838**	Pregnancy zone protein	Pzp	-0,58	0,00010109
**Q91W97**	Putative hexokinase HKDC1	Hkdc1	-0,58	0,04130176
**Q9CQ01**	Ribonuclease T2	Rnaset2a	-0,58	0,00060683
**O88967**	ATP-dependent zinc metalloprotease YME1L1	Yme1l1	-0,59	0,0011191
**P02469**	Laminin subunit beta-1	Lamb1	-0,59	0,00010643
**P20060**	Beta-hexosaminidase subunit beta	Hexb	-0,59	0,00010765
**Q3UW53**	Protein Niban	Niban1	-0,59	0,00510503
**Q6ZQ06**	Centrosomal protein of 162 kDa	Cep162	-0,59	0,0015103
**Q8BH98**	Lysophospholipid acyltransferase 1	Mboat1	-0,59	0,01225339
**Q8BND5-3**	Isoform 3 of Sulfhydryl oxidase 1	Qsox1	-0,59	0,00826216
**Q921C5-1**	Protein bicaudal D homolog 2	Bicd2	-0,59	0,00235005
**Q99KN1-1**	arrestin domain-containing protein 1	Arrdc1	-0,59	0,00013701
**Q9CQH0**	PDZK1-interacting protein 1	Pdzk1ip1	-0,59	0,00212119
**P16045**	Galectin-1	Lgals1	-0,6	0,00152865

**Q8JZM8**	Mucin-4	Muc4	-0,6	0,00888276
**Q8K0C5**	Zymogen granule membrane protein 16	Zg16	-0,6	5,7763E-05
**P35230**	Regenerating islet-derived protein 3-beta	Reg3b	-0,61	0,00362634
**P39061-3**	Collagen alpha-1(XVIII) chain	Col18a1	-0,61	0,02919074
**P52927**	high mobility group protein HMGI-C	Hmga2	-0,61	7,0641E-05
**Q921Z5-1**	Tumor necrosis factor alpha-induced protein 8	Tnfaip8	-0,61	0,00673481
**Q9R0Y5-1**	Adenylate kinase isoenzyme 1	Ak1	-0,61	7,4556E-05
**Q3TFQ1**	SPRY domain-containing protein 7	Spryd7	-0,62	0,02463351
**Q63886-1**	UDP-glucuronosyltransferase 1-1	Ugt1a1	-0,62	0,00080144
**Q8R3Q0**	Store-operated calcium entry-associated regulatory factor	Saraf	-0,62	0,04020737
**O08797**	SPI6	Serpinb9	-0,63	3,7821E-05
**Q8K2I4**	Beta-mannosidase	Manba	-0,63	0,0004509
**Q91W40**	Kinesin light chain 3	Klc3	-0,64	0,01431303
**P21447**	Multidrug resistance protein 1A	Abcb1a	-0,66	2,1502E-06
**P24452**	Macrophage-capping protein	Capg	-0,67	0,00016534
**Q60866**	Phosphotriesterase-related protein	Pter	-0,67	0,04193082
**Q8C0N2**	Glycerol-3-phosphate acyltransferase 3	Gpat3	-0,67	4,6111E-06
**P15626**	Glutathione S-transferase Mu 2	Gstm2	-0,68	6,0058E-06
**Q8BTV1**	Tumor suppressor candidate 3	Tusc3	-0,69	0,00012795
**F8VQM5**	Sucrase isomaltase (alpha-glucosidase)	Sis	-0,7	0,00234907
**Q9DC23**	DnaJ homolog subfamily C member 10	Dnajc10	-0,7	1,733E-06
**P31809**	Carcinoembryonic antigen-related cell adhesion molecule 1	Ceacam1	-0,71	0,00230646
**Q99N95**	39S ribosomal protein L3, mitochondrial	Mrpl3	-0,71	4,0859E-05
**P16406**	Glutamyl aminopeptidase	Enpep	-0,72	0,03502274
**Q4LDF6**	Complement factor H-related 2	Cfhr2	-0,72	5,4618E-05
**Q8BK48**	Pyrethroid hydrolase Ces2e	Ces2e	-0,72	6,7774E-05
**Q9JJV2-1**	Profilin-2	Pfn2	-0,72	0,00018859
**P07724**	Serum albumin	Alb	-0,73	4,8106E-07
**Q02496**	mucin-1	Muc1	-0,73	2,9533E-07
**Q80VQ0**	Aldehyde dehydrogenase family 3 member B1	Aldh3b1	-0,74	0,00014136
**E9Q236**	ATP-binding cassette, sub-family C (CFTR/MRP), member 4	Abcc4	-0,75	0,00217697
**Q06890**	Clusterin	Clu	-0,75	4,429E-07
**Q99MZ6**	Unconventional myosin-VIIb	Myo7b	-0,75	1,8391E-07
**P58802**	TBC1 domain family member 10A	Tbc1d10a	-0,76	0,0018521
**Q8VDC0**	Probable Leucine--tRNA ligase, mitochondrial	Lars2	-0,77	6,3794E-05
**Q9JKY7**	Cytochrome P450 CYP2D22	Cyp2d22	-0,78	1,3658E-07
**P23927**	Alpha-crystallin B chain	Cryab	-0,79	7,4223E-05
**Q8VHF2-1**	Cadherin-related family member 5	Cdhr5	-0,8	0,00088231
**Q9QXS6**	drebrin	Dbn1	-0,8	0,04081544
**P29391**	Ferritin light chain 1	Ftl1	-0,81	2,8488E-05
**Q00493**	Carboxypeptidase E	Cpe	-0,81	5,601E-09
**Q8BXK9**	Chloride intracellular channel protein 5	Clic5	-0,81	4,776E-06
**Q8R0I0-1**	angiotensin-converting enzyme 2	Ace2	-0,81	0,00060281
**O09043**	Napsin-A	Napsa	-0,82	0,00032958
**P31324**	cAMP-dependent protein kinase type II-beta regulatory subunit	Prkar2b	-0,82	0,00042614
**P16125**	L-lactate dehydrogenase B chain	Ldhb	-0,84	2,0377E-05
**Q8C025**	Cholinephosphotransferase 1	Chpt1	-0,84	2,7548E-09
**E9Q5I3**	Mucin 5, subtype B, tracheobronchial	Muc5b	-0,87	1,8923E-09

**O08992**	Syntenin-1	Sdcbp	-0,87	9,2238E-05
**Q6NZL0**	Protein SOGA3	Soga3	-0,88	0,03862916
**Q148W0**	Phospholipid-transporting ATPase IC	Atp8b1	-0,89	0,05163169
**F8VPQ6**	Alkaline phosphatase	Alpi	-0,9	5,0125E-05
**Q9D1G0**	Deoxyribonuclease-1-like 2	Dnase1l2	-0,9	6,4415E-07
**B5THE2**	Maltase-glucoamylase	Mgam	-0,91	0,00268891
**Q07456**	Protein AMBP	Ambp	-0,91	2,7901E-10
**Q08ED0**	Bcl-2-like protein 15	Bcl2l15	-0,93	9,8801E-12
**Q61581**	Insulin-like growth factor-binding protein 7	Igfbp7	-0,93	2,395E-11
**Q8BHL4**	Retinoic acid-induced protein 3	Gprc5a	-0,93	8,1936E-11
**Q8K3J9**	G-protein coupled receptor family C group 5 member C	Gprc5c	-0,93	2,8122E-05
**Q9D8W7**	OCIA domain-containing protein 2	Ociad2	-0,94	0,00206431
**Q9EP89**	Serine beta-lactamase-like protein LACTB, mitochondrial	Lactb	-0,94	2,8808E-05
**Q07797**	Galectin-3-binding protein	Lgals3bp	-0,96	8,8959E-10
**Q8JZQ5**	Amiloride-sensitive amine oxidase [copper-containing]	Aoc1	-0,96	1,5346E-11
**Q8QZR3-1**	Pyrethroid hydrolase Ces2a	Ces2a	-0,96	1,833E-11
**O08601**	microsomal triglyceride transfer protein large subunit	Mttp	-0,98	3,5922E-06
**Q75N73**	Zinc transporter ZIP14	Slc39a14	-0,98	3,3643E-06
**P13541**	Myosin-3	Myh3	-0,99	2,2118E-06
**P10649**	Glutathione S-transferase Mu 1	Gstm1	-1,01	1,0397E-12
**E9Q7P9**	Cadherin-related family member 2	Cdhr2	-1,02	1,2947E-06
**P43137**	Lithostathine-1	Reg1	-1,02	0,00040356
**O88338**	Cadherin-16	Cdh16	-1,04	3,0506E-15
**Q8K183**	Pyridoxal kinase	Pdxk	-1,04	3,2791E-09
**P11859**	Angiotensinogen	Agt	-1,08	2,2798E-07
**P21614**	vitamin D-binding protein	Gc	-1,12	7,7096E-12
**P09528**	Ferritin heavy chain	Fth1	-1,13	3,0506E-15
**Q3UW98**	Chloride channel accessory 4B	Clca4b	-1,16	3,0506E-15
**Q9D312**	Keratin, type I cytoskeletal 20	Krt20	-1,16	3,0506E-15
**Q9Z0L8-1**	Gamma-glutamyl hydrolase	Ggh	-1,18	5,7316E-14
**P55050**	Fatty acid-binding protein, intestinal	Fabp2	-1,21	7,9833E-13
**Q91Y97**	fructose-bisphosphate aldolase B	Aldob	-1,23	3,0506E-15
**O54990-1**	Prominin-1	Prom1	-1,28	3,0506E-15
**Q91X72**	Hemopexin	Hpx	-1,28	3,9071E-10
**E9PWP7**	Mucin 3, intestinal	-	-1,3	5,7316E-14
**Q9Z0J7**	Growth/differentiation factor 15	Gdf15	-1,46	3,0506E-15
**Q921I1**	Serotransferrin	Tf	-1,48	3,0506E-15
**Q61147**	Ceruloplasmin	Cp	-1,54	3,0506E-15
**Q91WL5**	Cytochrome P450 4A12A	Cyp4a12a	-1,55	3,0506E-15
**Q9Z0N2**	Eukaryotic translation initiation factor 2 subunit 3, Y-linked	Eif2s3y	-1,71	3,0506E-15
**Q3UZZ4**	Olfactomedin-4	Olfm4	-1,81	3,0506E-15
**E9Q035**	Predicted gene 20425	-	-1,84	3,0506E-15
**Q62011**	Podoplanin	Pdpn	-1,92	3,0506E-15
**Q62095**	atp-dependent rna helicase ddx3y	Ddx3y	-2	8,3909E-09
**Q8VCT4**	Carboxylesterase 1D	Ces1d	-2,13	3,0506E-15
**P10923**	Osteopontin	Spp1	-2,41	3,0506E-15

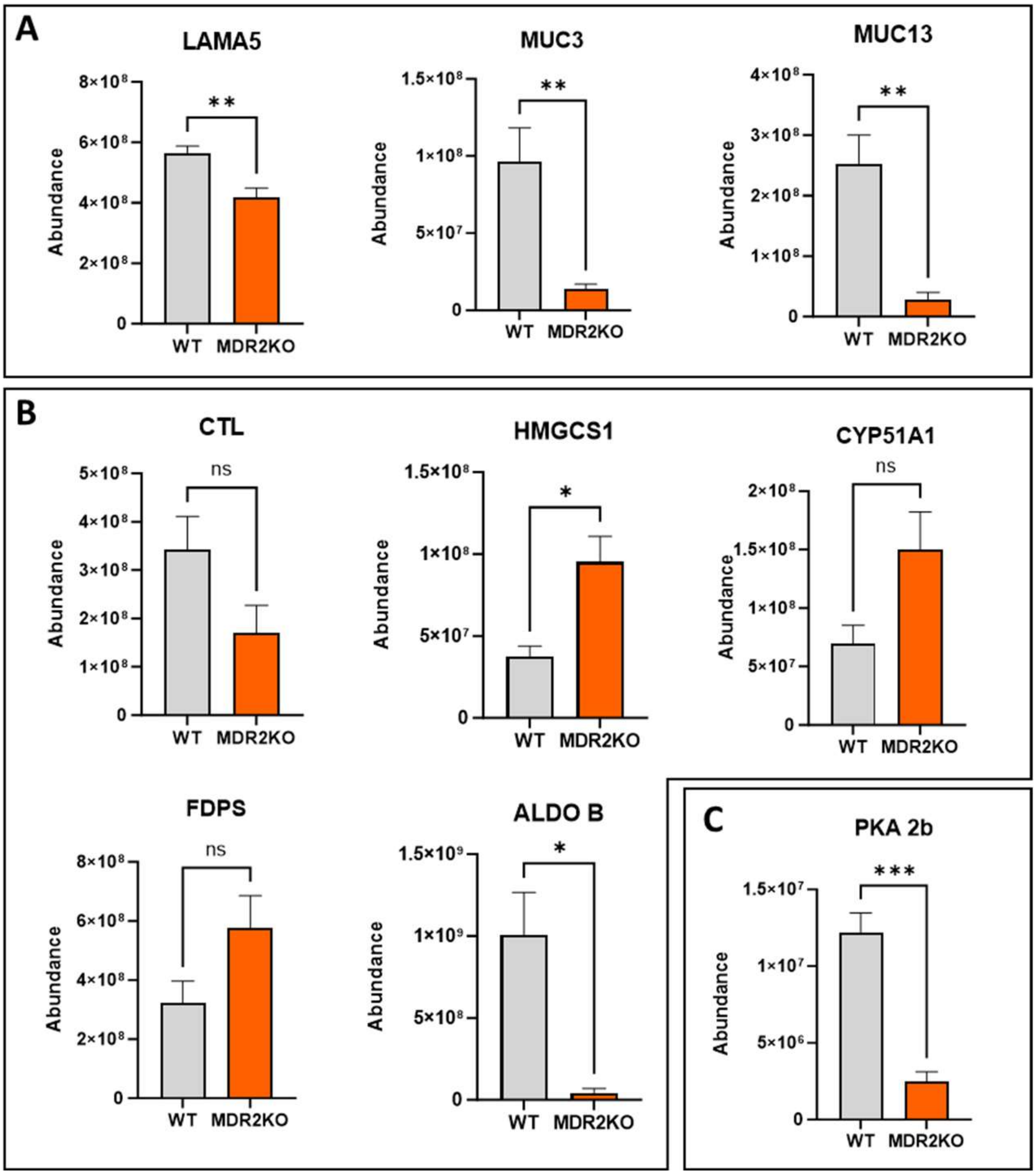

Supplementary figure 1. PRM MS analysis. (A) Representative proteins of alterations in the cell-ECM interactions (n=4). (B) Representative proteins of the rewiring of the intermediate metabolism (n=4). (C) Representative protein of the regulation of cell proliferation and differentiation (n=4).

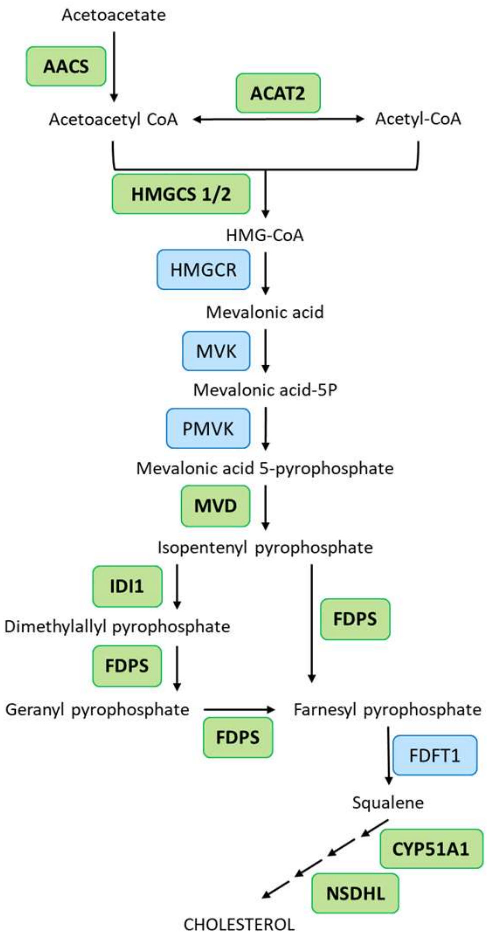

Supplementary figure 2. Schematic colesterol synthesis pathway. Differential up-regulated proteins identified in proteomic analysis are indicated in green.

A

Gen	Gene ID	Description	Forward primer (5' → 3')	Reverse primer (5' → 3')
PCK1	18534	Phosphoenolpyruvate carboxykinase	CACCATCACCTCCTGGAAGA	GGGTGCAGAATCTCGAGTTG
G6PC	14377	Glucose-6-phosphatase	GTGGCAGTGGTCGGAGACT	ACGGGCGTTGTCCAAAC

B

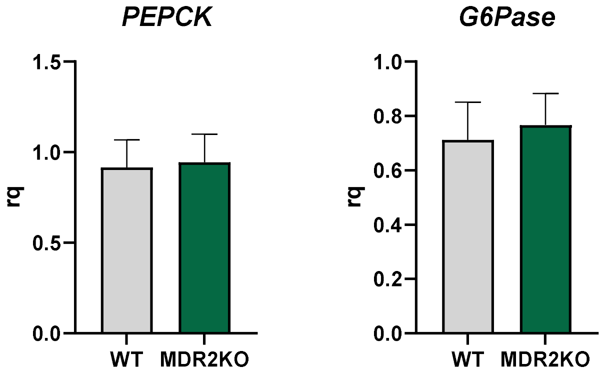

Supplementary figure 3. mRNA levels of FOXO1 target genes. (A) Primers used por RT-qPCR. (B) No change in FOXO1 target genes mRNA levels. Green: RT-qPCR (n=4).

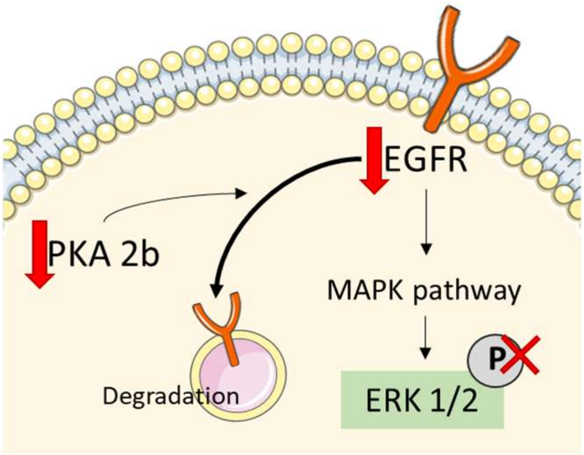

Supplementary figure 4. EGFR/PKA/ERK schematic pathway. Changes that have been observed in proteomic analysis are shown with red arrows and crosses.

WESTERN BLOT MEMBRANES

FIGURE 3C

pAKT

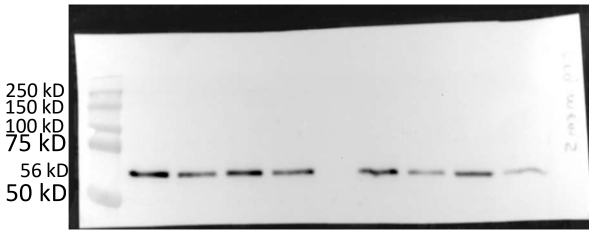

AKT

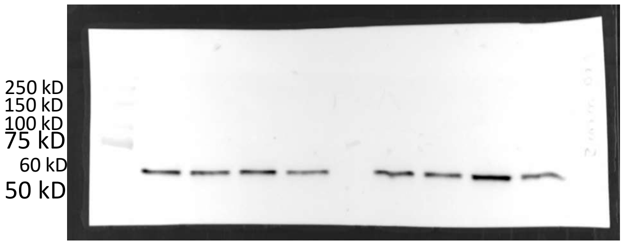

FIGURE 4D

pAMPK

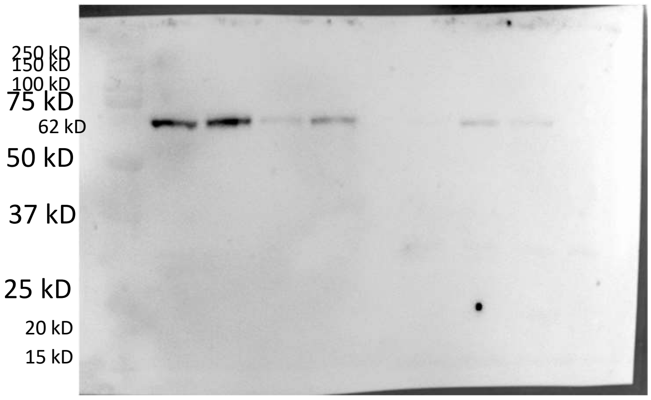

AMPK

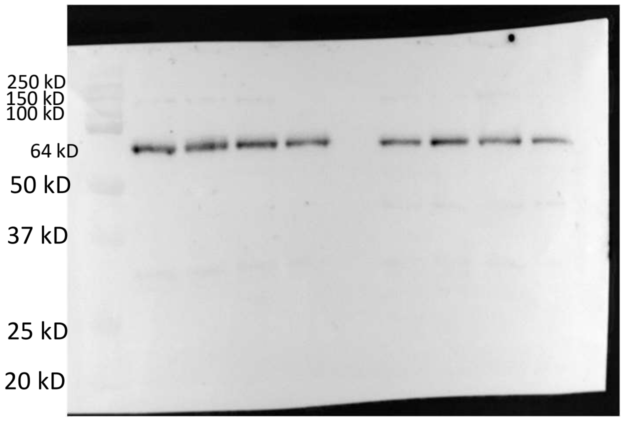

GAPDH

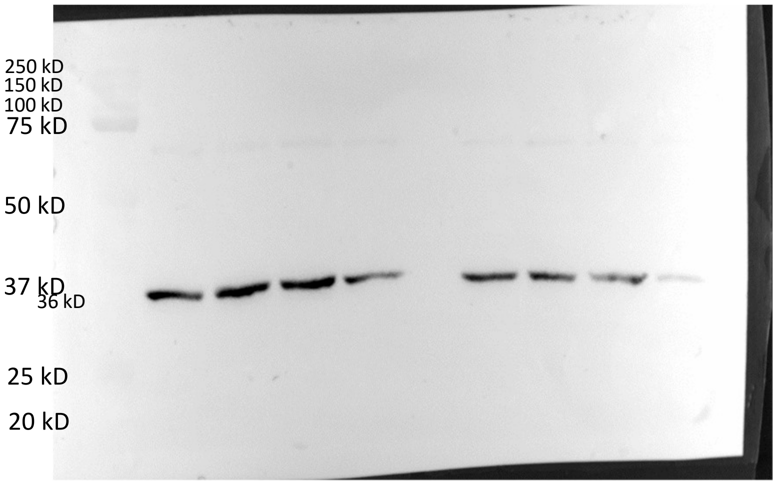

FIGURE 6C

pERK

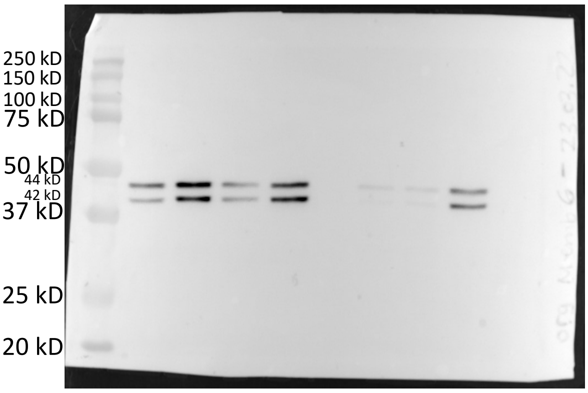

ERK

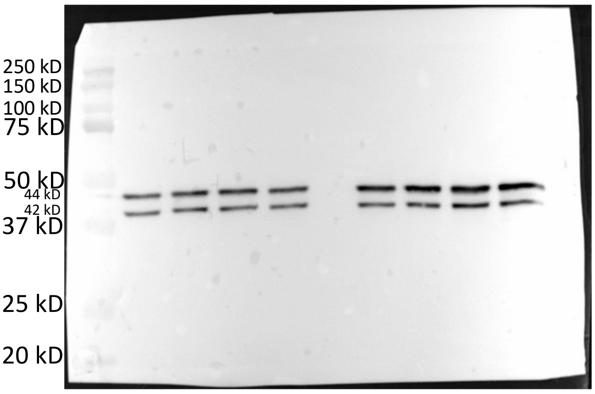

GAPDH

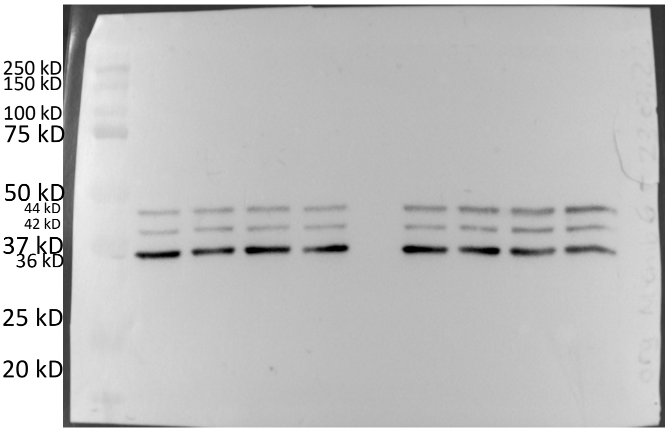

Supplement: Supplementary file 1 — pr3c00900_si_001.pdf [file pr3c00900_si_001.pdf]
